# Photobiomodulation accelerates orthodontic alignment in the early phase of treatment

**DOI:** 10.1186/2196-1042-14-30

**Published:** 2013-09-19

**Authors:** Chung How Kau, Alpdogan Kantarci, Tim Shaughnessy, Amornpong Vachiramon, Peerapong Santiwong, Alvaro de la Fuente, Darya Skrenes, Dennis Ma, Peter Brawn

**Affiliations:** Department of Orthodontics, School of Dentistry, University of Alabama, Birmingham, AL 35233 USA; Department of Applied Oral Health Sciences, Forsyth Institute, Cambridge, MA 02142 USA; Suwanee, GA USA; Bangkok, Thailand; Department of Orthodontics, Faculty of Dentistry, Mahidol University, Bangkok, 10400 Thailand; Montreal, Canada; Biolux Research Ltd, Vancouver, V6A 1H7 Canada; Data-Analysis.ca, Vancouver, Canada

**Keywords:** Orthodontic tooth movement, Photobiomodulation, Alignment, Accelerates

## Abstract

**Background:**

Numerous strategies have been proposed to decrease the treatment time a patient requires in orthodontic treatment. Recently, a number of device-accelerated therapies have emerged in orthodontics. Photobiomodulation is an emerging area of science that has clinical applications in a number of human biological processes. The aim of this study was to determine if photobiomodulation reduces the treatment time in the alignment phase of orthodontic treatment.

**Methods:**

This multicenter clinical trial was performed on 90 subjects (73 test subjects and 17 controls), and Little's Index of Irregularity (LII) was used as a measure of the rate of change of tooth movement. Subjects requiring orthodontic treatment were recruited into the study, and the LII was measured at regular time intervals. Test subjects used a device which produced near-infrared light with a continuous 850-nm wavelength. The surface of the cheek was irradiated with a power density of 60 mW/cm^2^ for 20 or 30 min/day or 60 min/week to achieve total energy densities of 72, 108, or 216 J/cm^2^, respectively. All subjects were fitted with traditional orthodontic brackets and wires. The wire sequences for each site were standardized to an initial round alignment wire (014 NiTi or 016 NiTi) and then advanced through a progression of stiffer arch wires unit alignment occurred (LII < 1 mm).

**Results:**

The mean LII scores at the start of the clinical trial for the test and control groups were 6.35 and 5.04 mm, respectively. Multi-level mixed effect regression analysis was performed on the data, and the mean rate of change in LII was 0.49 and 1.12 mm/week for the control and test groups, respectively.

**Conclusions:**

Photobiomodulation produced clinically significant changes in the rates of tooth movement as compared to the control group during the alignment phase of orthodontic treatment.

## Background

Every year, almost two million children in the USA receive orthodontic treatment, with an ever-increasing trend of adults seeking alignment of their teeth. Orthodontic therapy is predictable [1] where conventional methods result in the completion of treatment in 12 to 24 months and a variable follow-up period for the retention. One of the common deterrents to orthodontic treatment is the length of time in which a patient needs to commit. Thus, there has been a continuous search for methods to enhance the rate and efficacy of orthodontic tooth movement [2, 3]. At present, there are three main strategies to improve treatment efficiencies. The first approach is to create an accurate road map of the end point of orthodontic treatment. These treatment plans utilize sophisticated three-dimensional virtual plans to simulate and predict the possible pitfalls in a case [4]. Often, these provide the shortest pathway between the initial maligned tooth position and its final tooth position, providing an excellent visualization for the delivery of the best biomechanical plan and serving for patient education. A second approach involves the enhancement of mechanical aspects of tooth movement [2]. Conventional efforts to this end have been focused on enhancing the biomaterial properties and biomechanical interactions of orthodontic brackets and wires based on innovations regarding orthodontic wires and self-ligating systems [5]. Collectively, the progress has reduced the binding interactions of brackets and established constant force systems. Arguably, these enhancements have reached their peak, and any further advancement would result in a minimal impact on the length of orthodontic treatment.

A third approach aims to increase the rate of orthodontic tooth movement through biologically based techniques [6, 7]. One of the best characterized methods is surgical corticotomy-accelerated orthodontic tooth movement [8–11]. Clinicians raise surgical flaps around the dentoalveolar complex and create selective buccal and lingual decortications of the alveolar bone using rotary and hand instruments or piezoelectricity [12]. Active orthodontic treatment is applied almost immediately. While results from available data have been variable [13–16], the most important finding was that there is a window of intervention for accelerated tooth movement following surgical procedures. Once the wound resolution of the corticotomy sites is completed, the 'accelerated’ tooth movement returns to the rates of the control sites [17–19]. Surgery, even if it is highly effective and predictable, potentially carries the risk for morbidity and needs to be carefully planned with the orthodontic protocol and precisely timed for maximum effect during the course of treatment. In addition, beyond the clinical case series and anecdotal evidence, randomized clinical trials are required for an accurate assessment of the outcomes of surgical corticotomies in humans. Nonsurgical alternatives to the highly invasive surgical methods have been explored. Endothelial growth factors [20], osteoclast precursors like osteocalcin, prostaglandins [21], bone resorptive factors like RANKL [22], leukotrienes [23], and macrophage colony-stimulating factors have been tested. Studies in these areas are limited, which makes understanding these mechanisms difficult. Another recently explored area involves device-assisted therapy to biologically enhance the orthodontic tooth movement. To this end, a number of systems such as light, electrical currents [24], cyclic forces [25], and resonance vibration [26] have been introduced. This area is emerging while majority of these methods have been limited to case reports.

Light-accelerated orthodontics (LAO) is a technique within the scope of photobiomodulation or low-level light therapy (LLLT). The terms photobiomodulation and LAO can be interchangeably used to define the specific wavelength range, intensity, and light penetration and to differentiate from other methods utilizing light for treatment elsewhere in dentistry. LAO shows promise in producing a noninvasive stimulation of the dentoalveolar complex with a potential impact on ATP production by mitochondrial cells. The assumption is that an increase in ATP at a localized site will induce cells to undergo a remodeling process due to an elevated metabolic activity. Cytochrome oxidase c mediates ATP production. It is upregulated twofold by infrared light [27]. During the tooth movement phase, higher ATP availability helps cells 'turnover’ more efficiently leading to an increased remodeling process and accelerated tooth movement. LAO may also be functioning through an increased vascular activity [28], which would also contribute to the rapid turnover of the bone and is amenable to light [29]. A number of clinical case series have suggested an enhanced impact by LAO [2, 30], increased velocity of canine movement and decreased pain [31], and a significantly higher acceleration of retraction of treated canines [32]. However, there are also some studies that show questionable efficacy [33, 34]. There have been, however, no large-scale human studies correlating the use of an LAO device, which delivers low-level light therapy to the alveolus, and the rate of orthodontic tooth movement. The aim of this study was, therefore, to determine if a LAO device reduces treatment time in the alignment phase in a specific dental malocclusion by comparing the results to a similarly matched cohort of individuals. The null hypothesis of the study was that there was no difference in treatment effects as a result of photobiomodulation.

## Methods

### Subject recruitment and study groups

This is a multicenter clinical study. Subjects were recruited from four participating clinical sites as shown in Table 1. Each site received approval from their respective institutional review boards (University of Alabama at Birmingham) prior to the start of the trial. All subjects requiring orthodontic treatment and who met the following inclusion criteria were invited to participate in the study:

-  Permanent dentition
-  Patients who, in the opinion of the investigator, will be compliant with device use
-  Class I malocclusion with irregularity score [33] of >2 mm in either arch
-  Good oral hygiene

**Table 1:** **Demographics of the patient population**

	Number	Gender	Age (years)		
		%female	Mean	SD	Range
Center					
1	26	77	23	6	13 to 35
2	20	50	14	3	11 to 27
3	12	52	14	2	11 to 18
4	32	75	17	7	10 to 36
Total	90	69	18	7	10 to 36

The exclusion criteria were as follows:

-  Any medical or dental condition that could potentially affect study results
-  Patients currently using any investigational drug or any other investigational device
-  Patients planning to relocate or move during the treatment period
-  Use of bisphosphonates
-  Pregnant females

All subjects consented to participate in the study. Subjects were randomized into groups with varying treatment exposure times: (1) single exposure of 20 min/day, (2) single exposure of 30 min/day, and (3) single exposure of 1 h every week. The control group consisted of patients receiving conventional orthodontic treatment and with similar dental malocclusions.

### Orthodontic mechanics and clinical assessment of tooth movement

In this study, only the alignment phase of the orthodontic treatment was evaluated. Traditional orthodontic brackets and wires were used on all subjects. The wire sequences for each site were standardized to an initial round alignment wire (014 NiTi or 016 NiTi) and then advanced through a progression of stiffer arch wires unit alignment occurred (Little's Index of Irregularity (LII) < 1 mm). Outcome assessments were scheduled on a regular basis every 2 weeks for a 6-week period and then every 4 weeks until alignment was achieved. Tooth movement was assessed primarily by Little's Index of Irregularity for displacement [35]. LII was performed at baseline and each subsequent visit until LII was 1 mm or less. Measurements were made at the five contact points for the anterior teeth located between the canine teeth for each arch. The index recorded the displacements for each of the five points in millimeters. The change in tooth positions were recorded by designated calibrated operators at each site. In addition, clinical photographs representing the occlusal and buccal views of the dentition were obtained.

### Device description

**Figure 1:**
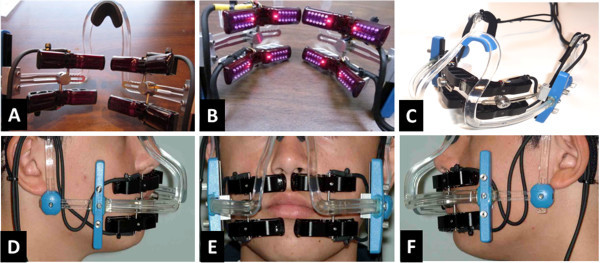
**Device components and a clinical presentation. (A**, **B)** A set of four extra-oral treatment arrays, each with a flexible printed circuit board and a set of LEDs mounted on a contoured heat sink and infrared-transmissible plastic lens, with conductive cables to the controller. **(C)** A headset similar to an eyeglass support structure to be worn by the patient on a daily or weekly basis, with attachment and adjustment mechanisms to position the treatment arrays in the appropriate location for the given patient. **(D**, **E**, **F)** Clinical presentation of the device.

Test subjects used a device (Extra-oral OrthoPulse LED, Biolux Research, Vancouver, Canada) which produced near-infrared light with a continuous 850-nm wavelength. The surface of the cheek was irradiated with a power density of 60 mW/cm^2^ for 20 or 30 min/day or 60 min/week to achieve total energy densities of 72, 108, or 216 J/cm^2^, respectively. Industry-standard light emitting diodes (LEDs) were used to produce the light, with arrays of emitters arranged in a series of treatment arrays to cover the target area of the alveolus of both the maxilla and mandible. A clinical presentation of the use as well as the components of the study device is depicted in Figure 1. The device consists of three main components:

-  A small handheld controller which houses the microprocessor, the menu-driven software, and the LCD screen. The controller is programmable by the investigator for the number of treatment sessions and the session duration. The user interface indicates to the patient the number of sessions completed and the remaining time in each session. The controller plugs into the power mains via a medically approved, UL-certified power supply.
-  A set of four extra-oral treatment arrays, each with a flexible printed circuit board and a set of LEDs mounted on a contoured heat sink and infrared-transmissible plastic lens, with conductive cables to the controller (Figure 1A,B)
-  A headset similar to an eyeglass support structure to be worn by the patient on a daily or weekly basis, with attachment and adjustment mechanisms to position the treatment arrays in the appropriate location for the given patient (Figure 1C)

All light treatments in this study were provided extra-orally with the device. Any heat generated as a by-product of light generation was monitored and maintained below thresholds of electro-medical device safety standards. The device monitors and records patient compliance by operating the treatment arrays only when the device is worn by the patient, and the investigator can obtain compliance data at each patient visit. The device is considered a class II medical device in the USA and Canada and a class 2a device in Europe. For the purpose of this study, using the device in the protocol was determined to be a nonsignificant risk by the respective institutional review boards. As explained above, compliance to the usage of the device was captured by an inbuilt microprocessor embedded within the controller. The processor recorded the number of minutes the device was activated and recorded the number of sessions the patient utilized the device.

### Intra-examiner calibration

To assess intra-examiner reliability between the four examiners, four duplicate sets of plaster models were randomly selected. LII was measured on the dental cast by each examiner. Following the lead of Shrout and Fleiss [36], the reliability of the measurements was investigated using calculated intra-class correlations for two-way mixed effects models specifying fixed effects for examiners and their LII measurements on the six plaster models. The analysis revealed strong reliability of measurements (ICC > 0.95).

### Statistics

Demographic information pertaining to sex, age, and ethnicity were taken to facilitate a conscientious statistical analysis. Arch type, treatment center, and treatment time were also accounted and controlled for the impact on the study outcomes. Several patient level variables were given codes and used in our regression analyses. Age was coded in years at the patient's first visit and was fixed over the entire treatment period. Ethnicity, a categorical variable with outcomes for Caucasian, African, Asian, and other ethnicity, was coded with Caucasian set as the reference group. When using a categorical variable, a reference category must be omitted from modeling to serve as the comparison group: any statistical significance pertaining to a particular ethnicity group would be interpreted as that group's difference to the reference group - in this case, whites. Arch type was controlled for by dummy coding mandible as 1 and maxilla as 0. Treatment center was coded as a categorical variable representing the four treatment centers; treatment center four was designated as the reference group in our analysis. Finally, our independent variable of interest, LAO treatment, was dummy coded 1 for test patients and 0 for control patients. LII score and treatment time varied at the longitudinal level and were coded continuously in millimeters and days, respectively. Due to the complexity of the data, multi-level mixed effects regression analysis with unstructured covariance (allowing for all variances and covariances to be distinct) was applied. This method allows us to account for the longitudinal and nested structure of our data while controlling for multiple independent variables. Stata version 12 was used to conduct analyses in this study.

## Results

In total, 90 subjects were recruited into the study, with 73 patients receiving conventional orthodontic treatment with LAO (test group) and 17 receiving only the orthodontic therapy (control group). The mean age of the subjects in the treatment groups were 20 and 17 years respectively, a difference which is not statistically significant (*p* value > 0.23). Table 1 highlights the demographic characteristics of the patients as well as the distribution across the four treatment centers. The upper bounds of the control group were used to define the limits of the data used in the study analysis. As a result, observations with initial LII greater than 15.5 mm were omitted from the analysis.

**Figure 2:**
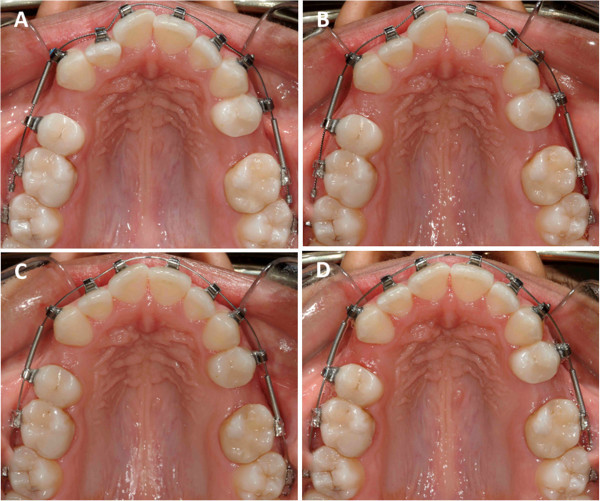
**A representative case treated with conventional orthodontic method in the control group. (A)** Baseline (day 0); LII is 3.80 mm. **(B)** Day 42; LII is 2.20 mm. **(C)** Day 119; LII is 1.70 mm. **(D)** Day 161; LII is 0.50 mm.

**Figure 3:**
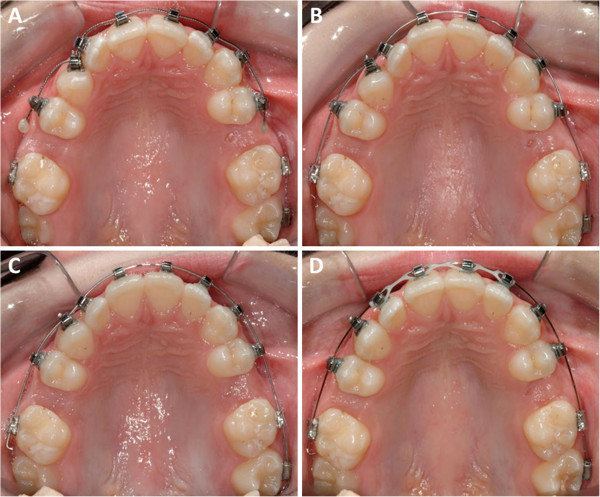
**A representative case treated with LAO method in the test group. (A)** Baseline (day 0); LII is 12.16 mm. **(B)** Day 14; LII is 9.30 mm. **(C)** Day 26; LII is 1.26 mm. **(D)** Day 56; LII is 0.00 mm.

**Figure 4:**
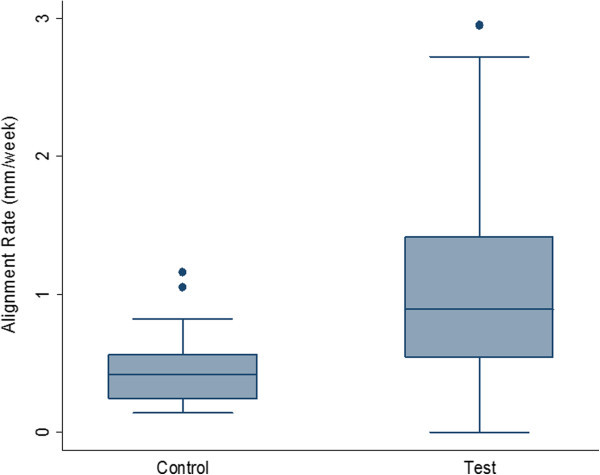
**Box plots showing differences in alignment rates (mm/week) between control and test (LAO) patients.** The box plots were created using arch level data to provide a more accurate weighting of alignment rates over total treatment time. Arch level summaries and Wilcoxon rank-sum tests revealed that the combined LAO arches started at a higher average LII (8.39 mm versus 6.67 mm). There was no statistically significant difference between the two groups in terms of destination LII. Outliers (rates greater than 3 mm/week) were removed from the test group to make these figures more conservative. The test group's mean alignment rates were 0.99 compared to a control rate of 0.44, with a comparison group of 23 control arches and 111 treatment arches.

Table 2 presents the differences in treatment outcomes and characteristics between the test and control groups. Representative cases for the control and test groups have been shown in Figures 2 and 3. The total number of data observations and summary statistics were calculated from time intervals. Wilcoxon rank-sum tests were conducted to assess whether or not the initial LIIs and overall alignment rates differed between the test and control groups. The *p* values associated with the tests are displayed below the means. The mean LII scores at the start of the clinical trial for the test and control groups were 6.35 and 5.04 mm, respectively. The mean rate of change in LII was 0.49 and 1.12 mm/week for the control and test groups, respectively (Figure 4). There was a statistically significant difference between study groups, indicating that subjects in the test group with LAO had a significantly faster rate of crowding resolution (*p* < 0.05).

**Table 2:** **Treatment outcome descriptive statistics comparing control to treatment group**

		Arches		Time intervals		Initial LII (mm)				Alignment rate (mm/week)			
	Number	Sample	Maxilla/mandible	Sample	Maxilla/mandible	Mean	SD	Median	Range	Mean	SD	Median	Range
Control	17	23	13/10	52	32/20	5.04	3.34	4.24	1.2 to 15.3	0.49	0.40	0.45	-0.15 to 1.55
Test	73	113	59/54	262	140/122	6.35	3.87	5.55	1.1 to 15.3	1.12	1.05	0.84	-0.19 to 7.49
*p* value								0.04				<0.00001

Table 3 displays the results of the multi-level mixed effects regression analysis. The table contains three models: the first establishes a baseline representation, the second appropriately characterizes the treatment time and lagged LII variables as nonlinear in form and interactive, and finally, the third phase in our variable of interest - the effect of LAO. As we control for lagged values for our dependent variable, models should be interpreted as being predictive of changes in LII.

**Table 3:** **Multi-level mixed effects linear regression models predicting Little's Irregularity Index**

	Model 1	Model 2	Model 3
Initial LII	0.599^*^	0.056	0.055
Female	0.241	0.257	0.317
Age	0.059^**^	0.051^**^	0.043
Ethnicity			
African	-0.798	-0.585	-0.609
Asian	-0.191	-0.167	-0.279
Other	-0.574	-0.651	-0.683
Mandible	-0.092	0.007	0.009
Test center			
Test center 1	-0.194	0.139	-0.008
Test center 2	0.025	0.349	-0.082
Test center 3	-0.275	-0.152	-0.183
Treatment time	-0.032^*^	-0.032^**^	-0.032^**^
LII (second)		0.048^*^	0.048^*^
Treatment time (second)		0.000^***^	0.000^***^
Treatment time × initial LII		-0.006^****^	-0.006^****^
Device			-0.881^***^
Constant	-0.749	0.591	1.584^**^
RI_Patient_Constant	0.089	-0.102	-0.133
RI_Arch_Constant	-0.550	-0.351	-0.358
RI_Res_Constant	0.483^*^	0.357^*^	0.355^*^
Chi square	508.554	716.377	730.174

NS: 314 time intervals nested within 136 arches nested within 90 patients. ^*^ *p* < 0.001, ^**^ *p* < 0.10, ^***^ *p* < 0.05, ^****^ *p* < 0.01.

Model 1 serves as a baseline model and highlights the significance of treatment time in predicting the LII. The regression analysis showed no statistically significant differences between treatment centers, gender, ethnicity, or arch (mandible or maxilla). Further inspection of the data suggested that LII and its relationship to treatment time were nonlinear in nature and should be examined interactively.

One notable change is that the second treatment center displays significantly worse alignment rates. These effects, however, are dispelled in Model 3 due to the fact that the contribution of control patients are not evenly distributed across treatment centers, that is, the second treatment center accounts for a larger proportion of control patients.

Model 3 introduces the LAO variable into our base model and balances out the treatment center effects of the previous model. Above and beyond all other factors controlled for, the LAO variable itself is significant at the 95% confidence level, providing strong evidence that LAO significantly improves the alignment rate. Finally, we noticed that a few treatment group records displayed remarkable alignment rates (>3 mm/week). We ran models without these observations and still found the results to be substantively unchanged.

## Discussion

The results demonstrate a significantly increased rate of tooth movement when photobiomodulation was used in conjunction with orthodontic treatment, suggesting that the biological impact of light accelerates orthodontic tooth movement in humans. A consecutive number of subjects were recruited from various study sites. While the randomization was individualized at each site, it was difficult to consistently apply to the entire sample. However, the study was conducted in a careful manner as possibly allowable.

A multi-level mixed effects linear regression to confidently control for a wide range of variables as well as subject-specific effects was employed in this study. Using this methodology, we were able to examine the possible effects of LAO above and beyond all other included variables. This approach allowed us to address the concern surrounding the difference in the amount of initial crowding between both groups in a way that a conventional *t* test or Mann–Whitney *U* test would not be able to do. The statistical evaluation demonstrated that there was no significant difference between centers. A major confounder that may potentially impact the outcome of multicenter trials is the variability of the measurements between different investigators. This was tested and investigators were calibrated using cast models and statistical methods. In order to adjust for other confounding factors, a multi-level mixed effects regression analysis with unstructured covariance was applied using the most obvious variables. This approach provided a comprehensive analysis of the data variables and added credibility to the study.

Previous studies have shown that the impact of the LLLT would be dependent on the wavelength and light intensity [37]. Light therapy at wavelengths of 660 nm produced a greater amount of bone mass around the teeth of rats. Another study showed that a laser-type semiconductor diode emitting infrared laser with a wavelength of 808 nm, 0.25 mW, and 10 s of exposure produced increased rates of tooth movement during the canine retraction phase of orthodontic treatment. The findings of increased rates of tooth movement corresponded to previously reported studies by Kawasaki et al. [38, 39]. In these studies, a similar rate of tooth movement was recorded in experimental rats. It was interesting to note that even higher rates of tooth movement were reported (2.08-fold) [40] when studying tooth movement in dogs over a 2-month period. In a further analysis of LLLT, investigators have suggested that a pulsed rather than constant method of light delivery produced better results [39]. The results of the current clinical trial not only confirm this increase in the rate of orthodontic tooth movement but also demonstrate its efficacy in humans in a large cohort. An important observation to note is that the impact of LAO is independent of the center and investigator effect, which makes it even more viable in clinical settings in general practice. Larger and longer clinical trials are required to assess the long-term stability of the treatment outcomes as well as the biological mechanisms.

Light and continuous forces applied to the dentoalveolar complex produce ideal rates of alignment. The efficiency of initial orthodontic treatment for the alignment of teeth has not been studied extensively in clinical trials with acceleration. One study reports the use of a three-dimensional measurement technique where LII was used to measure the rate of tooth movement [41]. Compared to previous reports [3, 42–44], the rate of alignment in the current study was substantially higher, where 1.12 mm/week of movement represented more than a 100% increase over the control group. One important aspect to note is that the initial LII was smaller in the patient groups in previous studies compared to our groups. It is unclear at this point whether the amounts of crowding and the greater need to overcome displacements of the initial malocclusion played an important role in the rates of tooth movement. This presents an important area for research to compare various modalities in literature. It is also important to note that higher initial LII scores were indicated in our test group, prompting us to utilize multiple regression techniques to control for any effects arising from variations in initial LII. As a result, any device effects in Table 3 occur above and beyond any initial LII effects. However, descriptive statistics for alignment rate, such as those found in Table 2, should be interpreted with the fact that the test and control groups have significantly different initial LII scores. It is entirely plausible that a positive correlation exists between LII scores and alignment rates. A cohort of control patients with higher initial LII scores could expect higher alignment rates, just as a cohort of test patients with lower initial LII scores could expect lower alignment rates. We are convinced, however, and supported by our regression model's findings that even with equivalent initial LII scores, a significant difference would be apparent between the control and test groups. Certainly, larger studies are warranted.

One other important aspect not evaluated in the study was the effectiveness and penetration of light from the test device. Energy densities (measured in Joules per square centimeter) have been reported to influence the rates of tooth movement. Investigators have suggested that lower energy density numbers have a more positive effect [45]. Due to the variability of facial structures (adipose tissue, skin texture, and bone densities of the jaws), it is difficult to effectively quantify the exact light exposure of the cells in the dentoalveolar complex.

Finally, an important area to consider is the normality of the cascade of events occurring during the tooth movement process. Any inherent defect in the genome relating to tooth movement will nullify the effects of LLLT in the system. This will lead to little or no tooth movement. In this study, no such defects relating to tooth movement were recorded. In the near future, orthodontists may combine 3D visualization with targeted orthodontic therapies, thus enhancing efficiencies through both a biological and mechanical approach. A substantial reduction in treatment will also potentially reduce the unwanted effects of orthodontic treatment, which include gingival recession, decalcification lesions on the surfaces of teeth, and patient enthusiasm for the treatment.

## Conclusions

The following may be concluded from this study:

-  Photobiomodulation produced clinically significant changes to the rates of tooth movement as compared to a control group during the alignment phase of orthodontic treatment, regardless of maxillary or mandibular arch.
-  The rates of tooth movement in the alignment phase were 1.12 mm/week for those in the photobiomodulation treatment group compared to 0.49 mm in the control group.
